# Ruptured Brain Aneurysm: A Rare Cause Of Isolated Acute Subdural Hematoma

**DOI:** 10.5334/jbsr.3748

**Published:** 2024-12-26

**Authors:** Simon-Pierre Docquier, Thomas Bonnet, Boris Lubicz

**Affiliations:** 1 Department of Interventional Neuroradiology, Erasme University Hospital, Brussels, Belgium

**Keywords:** Spontaneous acute subdural hematoma, ruptured aneurysms

## Abstract

*Teaching point:* Although computed tomography (CT) is the diagnostic gold standard for acute subdural hematoma, the absence of clear trauma should prompt the use of computed tomography angiography (CTA) to identify potential underlying causes, such as ruptured aneurysms, which can significantly influence treatment decisions.

## Case History

A 38‑year‑old woman presented to the emergency department with sudden‑onset right hemifacial pain persisting for 2 days, unrelieved by analgesics and accompanied by one episode of vomiting. There was no history of trauma, coagulopathy, infection, or arterial hypertension. The patient had a history of cocaine use and smoking. The neurological examination was unremarkable, and routine blood tests were within normal limits.

**Figure 1:**
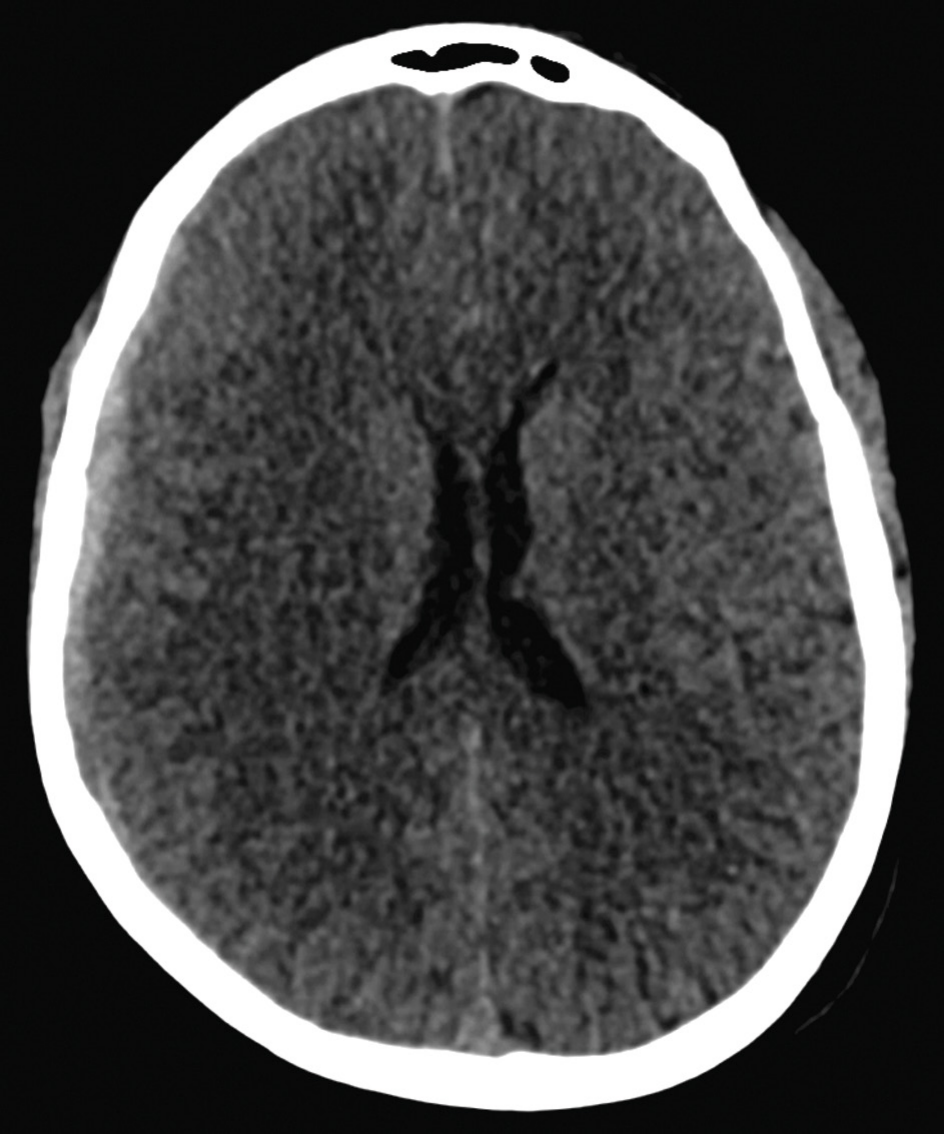
Non‑enhanced CT of the brain revealing a right hemispheric subdural hematoma.

A computed tomography (CT) of the brain revealed a right hemispheric subdural hematoma measuring 6 mm in thickness, extending to the right cerebellar tentorium and the interhemispheric fissure (Figure 1). There was limited mass effect on the right cerebral hemisphere, with a 4 mm midline shift to the left.

**Figure 2:**
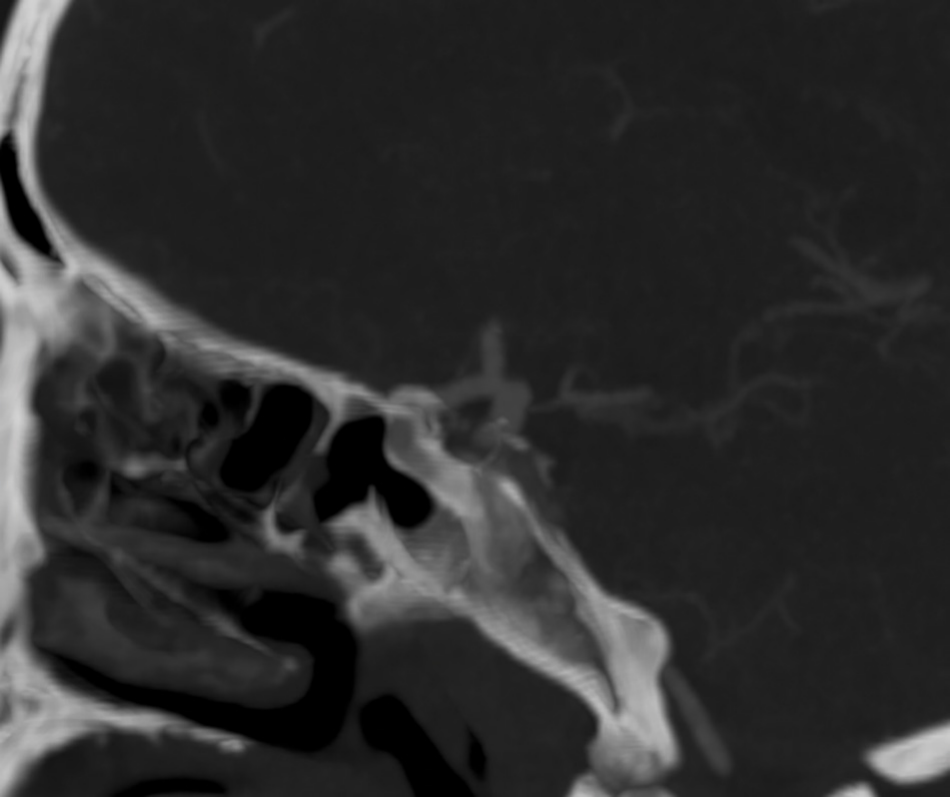
CT angiography revealing an irregular 10 mm aneurysm of the right posterior communicating artery.

Shortly thereafter, the patient’s condition deteriorated, exhibiting right‑sided ptosis, mydriasis and third nerve palsy. Given the clinical deterioration, a CT angiography (CTA) was performed, revealing an irregular 10 mm aneurysm of the right posterior communicating artery (Figure 2).

**Figure 3:**
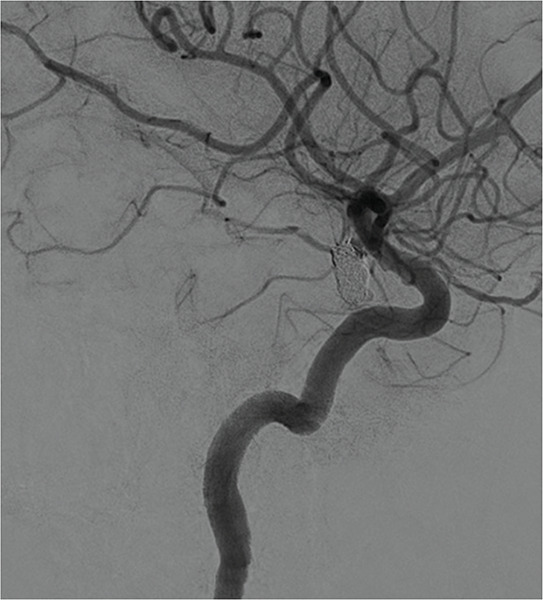
Lateral cerebral angiography confirming occlusion of the aneurysm.

Given the acute rupture, a simple coil embolization was successfully performed (Figure 3), and the patient was discharged in good clinical condition. A follow‑up MRI at 1 year confirmed complete resolution of the subdural hematoma and satisfactory occlusion of the aneurysm.

## Comments

While acute subdural hematoma (aSDH) is a common consequence of traumatic brain injury, spontaneous aSDH is a rare condition, with most literature consisting of case reports. The primary causes of spontaneous aSDH include ruptured aneurysms, leukemia, arteriovenous malformations, meningiomas, cerebral venous sinus thrombosis, neoplastic lesions, such as gliomas and metastases, and coagulopathies.

Several mechanisms have been proposed to explain how aneurysm rupture can lead to spontaneous aSDH: (1) massive, high‑pressure bleeding breaches the arachnoid membrane, spilling into the subdural space; (2) a compressive intracerebral hemorrhage tears the overlying cortex and arachnoid membrane; and (3) prior bleeding creates adhesions between the aneurysm sac and the arachnoid membrane, allowing subsequent rupture to bleed directly into the subdural space, as it might have been in our case.

Noncontrast enhanced CT remains the gold standard for diagnosing aSDH. However, in routine trauma settings, CTA is not typically performed. Absence of an obvious injury mechanism in case of aSDH should raise suspicion for an underlying aneurysm rupture, prompting the use of CTA, as identifying an underlying cause can significantly alter patient management.

Furthermore, when an aneurysm causes spontaneous aSDH, it is often associated with subarachnoid hemorrhage and/or intracerebral hematoma, helping radiologists in identifying the source of bleeding [1]. However, the absence of these findings, as in our case, should not preclude the use of CTA to detect an underlying lesion.
